# Sudden sensorineural hearing loss and viral association: serological evidence of herpesviruses and SARS-CoV-2^☆^

**DOI:** 10.1016/j.bjorl.2026.101909

**Published:** 2026-09-22

**Authors:** Tracy Lima Tavares Soeiro, Marina Cançado Passarelli Scott, Breno Lima de Almeida, Jônatas Bussador do Amaral, Elizabeth Cristina Perez, Sujana S. Chandrasekhar, Norma de Oliveira Penido

**Affiliations:** a Universidade Federal de São Paulo (UNIFESP), Escola Paulista de Medicina (EPM), Department of Otorhinolaryngology-Head and Neck Surgery, São Paulo, SP, Brazil; b Graduate Program in Environmental and Experimental Pathology, Paulista University, São Paulo, Brazil; c ENT and Allergy Associates, LLP, New York, United States of America; d ENT Research Lab, Department of Otorhinolaryngology Head and Neck Surgery, Universidade Federal de São Paulo (UNIFESP), São Paulo, Brazil

**Keywords:** Sudden sensorineural hearing loss, Herpesvirus, SARS-CoV-2, COVID-19, Prognosis

## Abstract

**Objective:**

To investigate the association between Herpesviruses (HSV-1, HSV-2, VZV, EBV, CMV) and SARS-CoV-2 in the acute phase of Sudden Sensorineural Hearing Loss (SSNHL), correlating serological and PCR findings with clinical and audiometric outcomes.

**Methods:**

Prospective cohort study including 53 patients evaluated between March 2020 and February 2024. Audiological assessment, serological testing (IgM and IgG), PCR for herpesviruses and SARS-CoV-2, and magnetic resonance imaging were performed.

**Results:**

Most patients (90.6%) presented with unilateral hearing loss. Serological evidence of viral exposure was observed in 43.4% of cases, including SARS-CoV-2 (26.4%), HSV (22.6%), and CMV (5.6%). Coinfection with two viruses occurred in 11.3% of patients and was associated with poorer hearing outcomes (p = 0.0032). Bilateral hearing loss (9.4%) was also associated with no improvement (p = 0.0368). In multivariate analysis, COVID-19 infection remained independently associated with poorer outcomes.

**Conclusion:**

SSNHL was associated with a high frequency of viral serological markers. Viral coinfection may be associated with poorer prognosis, although causality cannot be established. Initial hearing loss severity and bilateral involvement remained important prognostic factors. Serological findings may serve as complementary tools for clinical characterization and prognostic assessment, although their etiological role remains uncertain.

**Level of Evidence:**

2.

## Highlights

• High seropositivity rates for herpesviruses and SARS-CoV-2 were observed in patients with SSNHL.
• Viral coinfection was associated with poorer hearing outcomes.
• Severe initial hearing loss and bilateral involvement were associated with worse prognosis.
• Intralabyrinthine MRI signal abnormalities were more frequently observed in patients with positive viral markers.
• Serological findings may contribute to clinical characterization and prognostic assessment in SSNHL.

## Introduction

Sudden Hearing Loss (SHL) is defined as an abrupt reduction in hearing, occurring unilaterally or bilaterally within 72 hs, and is often accompanied by tinnitus and/or vertigo.^1^^,^^2^ It may present as sensorineural, mixed, or conductive, depending on the site and nature of auditory dysfunction. The most common form, Sudden Sensorineural Hearing Loss (SSNHL), is classically defined as a ≥30 dB loss across three consecutive audiometric frequencies within 72-hs, according to the National Institute on Deafness and Other Communication Disorders (NIDCD).^3^ Its incidence ranges from 5 to 27 cases per 100,000 individuals annually, with approximately 66,000 new cases reported each year in the United States. Bilateral association remains rare, occurring in only 0.4%–4.9% of cases.^3^, ^4^, ^5^

Despite being considered an otological emergency, the etiology of SSNHL remains unknown in nearly 70%–90% of patients and is thus classified as idiopathic.^3^^,^^6^ Several mechanisms have been proposed, including vascular, autoimmune, and infectious pathways.^6^^,^^7^ Among infectious agents, members of the Herpesviridae family ‒ such as HSV-1, HSV-2, VZV, EBV, and CMV ‒ have been extensively investigated due to their ability to establish latency and reactivate, potentially affecting inner ear structures including the cochlear nerve and the organ of Corti.^7^, ^8^, ^9^, ^10^

More recently, SARS-CoV-2, the causative agent of COVID-19, has been implicated as a possible trigger of SSNHL. Reports of otoneurological manifestations associated with COVID-19 have highlighted sudden hearing loss as a potential complication, although the underlying mechanisms remain unclear.^11^

Direct identification of viral involvement in the inner ear is limited due to the invasiveness of cochlear biopsy, making indirect methods such as PCR and serological testing essential for etiological investigation.^7^, ^8^, ^9^ Given the high proportion of idiopathic cases, viral infections may be underdiagnosed in clinical practice.

Therefore, this study aimed to investigate the presence and clinical impact of Herpesviridae and SARS-CoV-2 association, in patients with sudden sensorineural hearing loss, characterizing their audiometric and clinical profiles to advance etiological understanding and guide management strategies.

## Methods

### Study design and inclusion criteria

This was a prospective, single-center cohort study conducted between March 2020 and February 2024 at a tertiary referral outpatient clinic specialized in otology. All consecutive patients presenting with sudden sensorineural hearing loss during the study period were screened for eligibility to reduce selection bias and ensure representative inclusion.

Sudden Sensorineural Hearing Loss (SSNHL) was defined as a sensorineural hearing loss of at least 30 dB in three consecutive frequencies, confirmed by pure-tone audiometry.

Inclusion criteria were: (1) Age between 10- and 90-years; (2) Presentation within 30-days from symptom onset; (3) Diagnosis of SSNHL confirmed by audiometry; and (4) Completion of the standardized diagnostic workup, including audiometric evaluation and viral serology.

Exclusion criteria were: (1) Inability to complete follow-up or key components of the diagnostic protocol; or (2) Refusal to participate.

All participants provided written informed consent. The study protocol was approved by the institutional Research Ethics Committee (Approval 7.311.262), in accordance with the Declaration of Helsinki.

### Audiometric assessment

Confirmation of the diagnosis was based on the AAO-HNS criteria, employing the strict definition of sudden sensorineural hearing loss, with a threshold reduction of 30 dB or more, in at least three contiguous audiometric frequencies, occurring over a 72 h time window.^3^ An Interacoustics AD28, calibrated annually, was used to test airway hearing thresholds at 250 Hz–8000 Hz, bone conduction at 500 Hz–4000 Hz, Speech Audiometry, Speech Recognition Threshold (SRT) and Word Recognition Score (WRS). The masking used was Speech Noise and Narrow Band. Tympanometry analysis was also carried out.

Audiometric testing was performed at the time of initial diagnosis, followed by weekly evaluations during the first month of corticosteroid therapy and then at biweekly intervals. For the purposes of this study, the Pure Tone Average (PTA) was calculated using air conduction thresholds of 500, 1000, 2000 and 4000 Hz at onset and the final audiometry performed 60–90 days after onset were used to classify patients. The 4-frequency Pure Tone Average (4fPTA) value for bilateral cases was calculated by taking the arithmetic mean between the two affected ears. Hearing Loss (HL) was measured according to the World Health Organization (WHO) classification^12^: Normal hearing thresholds <20 dB, mild HL thresholds between 20 and 35 dB, moderate HL thresholds between 35 and 50 dB, moderately severe HL thresholds between 50 and 65 dB, severe HL thresholds between 65 and 80 dB, profound HL thresholds between 80 and 95 dB, and complete hearing loss thresholds >95 dB. Functional hearing was defined as 4fPTA thresholds ≤50 dB or WRS ≥50%.^12^^,^^13^

### Hearing outcomes classification

Hearing recovery was assessed by comparing the initial audiometric evaluation at presentation with the final audiometry performed 60–90 days after symptom onset, using a combination of 4fPTA and WRS.^14^

Previous audiometric data were limited; however, no patient had documented chronic hearing loss prior to the episode.

Outcomes were classified into two categories: (1) Improvement and (2) No improvement.

Improvement was defined as the presence of any clinically meaningful hearing recovery, including at least one of the following:

• An increase of ≥10 dB in 4fPTA and/or ≥10% in WRS;
• Recovery to functional hearing, defined as 4fPTA ≤50 dB and/or WRS ≥50%;
• Return to near-normal hearing, defined as a final 4fPTA within 10 dB of the contralateral ear in unilateral cases, or return to pre-morbid thresholds when previous audiograms were available.

No improvement was defined as:

• <10 dB improvement in 4fPTA and <10% change in WRS; or
• Persistence of non-functional hearing (4fPTA >50 dB and WRS <50%), even in the presence of minor audiometric changes; or
• Any deterioration in hearing thresholds during follow-up.

### Imaging and laboratory testing

Brain and temporal bone Magnetic Resonance Imaging (MRI) with gadolinium was performed in a subset of patients, according to clinical indication, primarily to exclude retrocochlear pathology, particularly cerebellopontine angle tumors, as well as other structural abnormalities. MRI was generally performed after the first month of symptom onset and, in some cases, during corticosteroid therapy.

A standardized 1.5-Tesla protocol was used, without dedicated endolymphatic hydrops sequences. The protocol included axial and coronal T1-weighted sequences (pre- and post-contrast), T2-weighted sequences, and Diffusion-Weighted Imaging (DWI).

Blood samples were collected at two time points: at presentation (within 30-days of onset) and at follow-up (60–90 days after onset). Serological testing for HSV-1, HSV-2, VZV, EBV, CMV, and SARS-CoV-2 (IgM and IgG) was performed using commercially available Enzyme-Linked Immunosorbent Assay (ELISA) kits. Viral PCR assays for the same pathogens were conducted following standardized protocols provided by the manufacturers. These evaluations aimed to identify active or recent viral infections potentially associated with SSNHL.

### Definitions of viral status

To ensure methodological transparency and alignment with current virological standards, serological and PCR results were classified as follows:

• Active infection or viral reactivation was defined as IgM-positive or PCR-positive with IgG-positive;
• Primary infection was defined as IgG-negative with either IgM-positive or PCR-positive;
• Coinfection was defined as IgM or PCR positivity for two or more viruses simultaneously, regardless of IgG status.

Given the known limitations of serological assays, including false-positive IgM results, cross-reactivity, and persistence or reappearance of IgM antibodies, the classification of “reactivation” should be interpreted with caution.

PCR detection in peripheral blood was included when available and interpreted as supportive evidence of viral activity; and results were analyzed in conjunction with serological findings.

All serological tests were performed using ELISA, according to manufacturer specifications, and interpreted based on established cutoff values.

### Treatment

Patients received standardized oral corticosteroid therapy at 1 mg/kg/day (maximum 60 mg) for up to 2-weeks, followed by a tapering. No patients received intratympanic corticosteroid injections during the study period. Supportive care, including symptom management for tinnitus or vertigo, was provided as clinically indicated.

### Statistical analysis

Descriptive statistics were used to summarize demographic, clinical, audiometric, and laboratory characteristics of the cohort. Associations between viral serology, PCR results, MRI findings, the laterality of Sudden Sensorineural Hearing Loss (SSNHL), and clinical outcomes were evaluated using exact Chi-Square tests and Cochran-Armitage trend analyses.

To identify factors associated with no improvement, multivariate analysis was performed using Poisson regression with robust variance, estimating Prevalence Ratios (PR) and their respective 95% Confidence Intervals (95% CI).

Variables with p < 0.25 in univariate analysis, along with clinically relevant covariates (including age group, sex, initial SSNHL classification at first consultation, comorbidities, associated symptoms, time to auditory recovery, and viral PCR and serological positivity), were included in the multivariate model. A stepwise approach was applied, and variables with p < 0.05 were retained in the final model.

Multicollinearity was assessed using tolerance values, with a cutoff of > 0.60. All statistical analyses were performed using SAS® software, version 9.4 (SAS Institute Inc., Cary, NC, USA), and statistical significance was set at p < 0.05.

## Results

A total of 67 patients were initially screened for eligibility. Fourteen patients were excluded based on predefined criteria, resulting in a final sample of 53 patients. Among the included patients, 56.6% were male, with a mean age of 46-years (Table 1). Most cases were unilateral (90.6%). Associated symptoms were frequent: tinnitus was reported by 90.6% of patients, while vertigo was present in 52.8%.

**Table 1:** Demographic and clinical profile of patients with SSNHL.

Variables	Frequency	Percentage
Gender		
Female	23	43.40%
Male	30	56.60%
Age Group		
10‒19	3	5.66%
20‒29	5	9.43%
30‒39	10	18.87%
40‒49	13	24.53%
50‒59	12	22.64%
60‒69	7	13.21%
70‒79	3	5.66%
Comorbidities		
Hypertension	21	39.62%
Diabetes	10	18.87%
Hypertension and diabetes (combined)	9	16.98%
Others	11	20.75%
None	20	37.74%
Associated Symptoms		
Absence	4	7.55%
Tinnitus	20	37.74%
Tinnitus and Dizziness	28	52.83%
Peripheral Facial Paralysis	1	1.89%

Hypertension (39.62%) and diabetes mellitus (18.87%) were the most prevalent comorbidities in the cohort. A considerable proportion of patients presented both conditions simultaneously (16.98%). Other less frequent comorbidities included dyslipidemia, asthma, hypothyroidism, autoimmune diseases, anxiety disorders, and chronic kidney disease.

Initial hearing loss severity was classified as normal in 9.4%, mild in 13.2%, moderate in 20.8%, severe in 30.2%, and profound in 26.4% of cases (Table 2). Overall, 66% of patients experienced some degree of clinical improvement during follow-up.

**Table 2:** Distribution of patients with SSNHL according to the degree of hearing loss in the first consultation and clinical evolution at the end of treatment.

Classification of Sudden Hearing Loss in First Consultation	Frequency	Percentage
Normal^a^	5	9.43%
Mild	7	13.21%
Moderate	11	20.75%
Severe	16	30.19%
Profound	14	26.42%
Improvement	35	66.04%
No improvement	18	33.96%

a The “Normal” classification includes cases with SSNHL in high frequencies, characterized by ramp loss. The mean 4fPTA of the tonal thresholds is within normal limits.

Serological testing revealed evidence of acute or recent viral exposure in 43.4% of patients. IgM positivity was observed for SARS-CoV-2 in 26.4% and for HSV in 22.6%. CMV IgM positivity was present in 5.6% (Table 3). All patients underwent PCR testing, which was negative for HSV, EBV, VZV, and SARS-CoV-2; only one patient tested positive for CMV (1.89%). Notably, IgG levels were elevated for VZV in 37.7% of patients (titers >1:100) and for EBV in 34% (titers >1:160), indicating past exposure or reactivation. Coinfection with two viruses occurred in 11.3% of patients, all of whom showed no clinical improvement (p = 0.0032, Table 4), highlighting the negative impact of viral overlap on prognosis.

**Table 3:** Distribution of patients by viral infection and association with clinical evolution.

Variables	Improvement	No Improvement	Total
COVID-19 IgM			
Negative	30 (85.71%)	9 (50.00%)	39 (73.58%)
Positive	5 (14.29%)	9 (50.00%)	14 (26.42%)
Herpes Simplex 1 & 2 IgM			
Negative	30 (85.71%)	11 (61.11%)	41 (77.36%)
Positive	5 (14.29%)	7 (38.89%)	12 (22.64%)
Cytomegalovirus IgM			
Negative	33 (94.29%)	17 (94.44%)	50 (94.34%)
Positive	2 (5.71%)	1 (5.56%)	3 (5.66%)

*Values are expressed in frequency (%).

**Table 4:** Distribution of patients according to viral coinfection and clinical evolution.

Viral Coinfection	Improvement	No Improvement	Total	p-value
None	23 (76.67%)	7 (23.33%)	30 (56.60%)	0.0032
One type of virus	12 (70.59%)	5 (29.41%)	17 (32.07%)	
Two types of viruses	0 (0.00%)	6 (100.00%)	6 (11.32%)	

*Values are expressed in frequency (%).

#p-value obtained by exact Cochran-Armitage trend test.

Brain and temporal bone MRI were performed in 39 of the 53 patients. The main indication was to exclude retrocochlear pathology, particularly cerebellopontine angle tumors. Fourteen patients did not undergo MRI due to clinical or logistical constraints.

The MRI protocol did not include dedicated sequences for the evaluation of endolymphatic hydrops due to limited availability.

Abnormal findings were observed in 35.8% of patients. The predominant findings consisted of intralabyrinthine signal alterations, typically hyperintense on T2-weighted and FLAIR sequences, occasionally with contrast enhancement on T1-weighted imaging, as illustrated in Fig. 1.

**Fig. 1:**
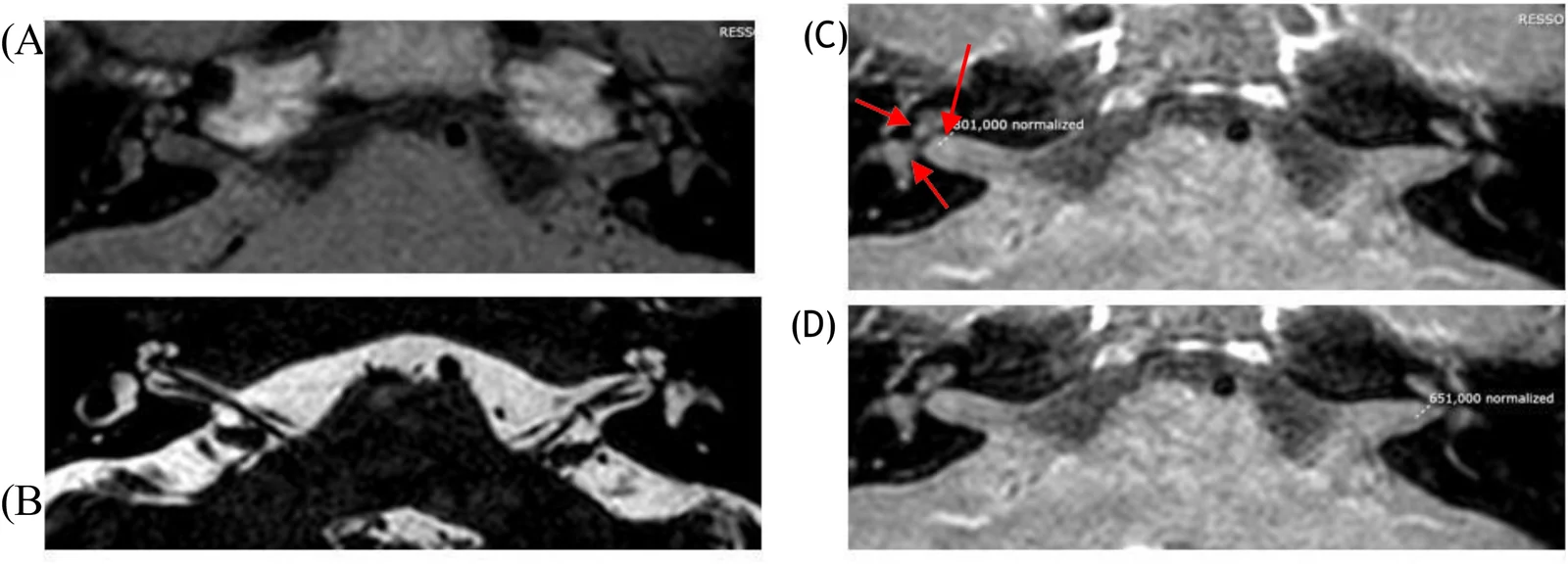
Temporal bone magnetic resonance imaging in axial sections. Source: Authors’ personal archive.

The images include: (A) T1-weighted sequence without contrast, showing no pathological hyperintensity in the inner ear structures; (B) T2 FIESTA/CISS sequence, highlighting the inner ear structures, semicircular canals, and vestibulocochlear nerve with high fluid resolution; (C) Post-contrast T1-weighted sequence with fat suppression, showing moderate enhancement, consistent with an inflammatory process (801,000 normalized) in the internal acoustic meatus, basal turn of the cochlea, and right vestibule (red arrows); (D) Post-contrast T1-weighted sequence with fat suppression, showing reduced signal intensity (651,000 normalized), indicating absence of lesion or low inflammatory activity on the left.

Regarding prognostic factors, bilateral hearing loss was significantly associated with poorer outcomes (p = 0.0368, Table 5). Initial severity remained the strongest predictor of non-recovery (p = 0.0173).

**Table 5:** Relationship between the side of sudden hearing loss and clinical evolution.

Side of Sudden Hearing Loss	Improvement	No Improvement	p-value
Right	15 (78.95%)	4 (21.05%)	0.0368
Left	19 (65.52%)	10 (34.48%)	
Bilateral	1 (20.00%)	4 (80.00%)	

*Values are expressed in frequency (%).

#p-value obtained by exact Chi-square test.

In the univariate analysis, COVID-19 (PR = 2.79; 95% CI 1.39–5.57; p = 0.0038), herpes simplex virus types 1 and 2 (PR = 2.17; 95% CI 1.08–4.36; p = 0.0287), time to auditory recovery (PR = 18.00; 95% CI 2.68–120.92; p < 0.0001), sex (PR = 1.99; 95% CI 0.83–4.79; p = 0.1231), and time from symptom onset to treatment (PR = 2.45; 95% CI 1.21–4.96; p = 0.0128) were associated with the outcome at p < 0.25 and were included in the multivariate model.

In the adjusted analysis, COVID-19 infection remained independently associated with a higher prevalence of no improvement of hearing, corresponding to a 111% increase compared to COVID-19-negative patients (p = 0.0129).

Time to auditory recovery, analyzed as a clinical course variable (<15 vs. ≥15 days), was also associated with the outcome, with a 10.74-fold higher prevalence of no improvement compared to patients who recovered within 15 days (p = 0.0158). No significant difference was observed between patients with recovery times longer than 15-days and those with recovery within 15-days (p = 0.5015, Table 6).

**Table 6:** Factors associated with no improvement in patients with sudden sensorineural hearing loss based on crude and adjusted prevalence ratios.

	Crude Prevalence Ratio (PR)		Adjusted Prevalence Ratio (PR) a	
Variables	PR (95% CI)	p-value	PR (95% CI)	p-value
COVID-19		0.0038		0.0129
Negative	1	‒	1	‒
Positive	2.79 (1.39; 5.57)	0.0038	2.11 (1.17; 3.81)	0.0129
Herpes simplex virus 1 and 2 ‒ IgM		0.0287	‒	‒
Negative	1	‒	‒	‒
Positive	2.17 (1.08; 4.36)	0.0287	‒	‒
Cytomegalovirus – IgM		0.9812	‒	‒
Negative	1	‒	‒	‒
Positive	0.98 (0.19; 5.09)	0.9812	‒	‒
Varicella-zoster virus (antibody levels)	1.00 (1.00; 1.00)	0.8847	‒	‒
Time to auditory recovery		<0.0001		0.0008
≤15 days	1	‒	1	‒
>15 days	2.57 (0.29; 22.61)	0.3945	1.93 (0.28; 13.21)	0.5015
No recovery	18.00 (2.68; 120.92)	0.0029	10.74 (1.56; 73.83)	0.0158
Associated symptoms		0.4305	‒	‒
Present	1	‒	‒	‒
Absent	1.53 (0.53; 4.42)	0.4305	‒	‒
Sex		0.1231	‒	‒
Female	1	‒	‒	‒
Male	1.99 (0.83; 4.79)	0.1231	‒	‒
Age group		0.7549	‒	‒
≤50 years	1	‒	‒	‒
>50 years	1.13 (0.53; 2.39)	0.7549	‒	‒
Time from symptom onset to treatment		0.0128	‒	‒
≤15 days	1	‒		
>15 days	2.45 (1.21;4.96)	0.0128		
Initial hearing loss severity		0.0460		0.1349
Normal/Mild/Moderate	1	‒	1	‒
Severe/profound	2.68 (1.02; 7.07)	0.0460	1.51 (0.88; 2.60)	0.1349
Comorbidities		0.2256		0.3432
None	4.50 (0.67; 30.06)	0.1206	2.23 (0.52; 9.63)	0.2809
Hypertension and diabetes (combined)	1	‒	1	‒
Hypertension or diabetes	1.64 (0.17; 15.26)	0.6655	1.18 (0.29; 4.83)	0.8219
Others	3.46 (0.48; 24.87)	0.2171	1.67 (0.41; 6.76)	0.4733

a Adjusted for COVID-19, time to auditory recovery, initial hearing loss severity and comorbidities.

## Discussion

This study explores the potential role of viral association in the pathogenesis and prognosis of Sudden Sensorineural Hearing Loss (SSNHL), as supported by high seropositivity rates for herpesviruses and SARS-CoV-2. Coinfection appeared to be associated with poorer hearing outcomes, raising the possibility of a synergistic effect of multiple viral agents in aggravating auditory damage.^15^^,^^16^ Given the sample size, this finding should be interpreted cautiously.

Bilateral involvement, although uncommon,^17^^,^^18^ was consistently associated with a lack of hearing recovery, in line with previous reports. Similarly, the degree of initial hearing loss remained the most important prognostic factor, corroborating prior studies and underscoring the need for early recognition and prompt management.^14^

In the multivariate analysis, COVID-19 infection remained independently associated with poorer hearing outcomes, reinforcing the potential role of viral factors in disease progression.

Delayed auditory recovery was also associated with worse outcomes, likely representing a marker of disease severity and clinical course rather than an independent predictor. Other variables did not retain statistical significance after adjustment, suggesting that their effects may be influenced by confounding factors.

Our findings contribute to the current body of knowledge by documenting the frequency of viral serological and molecular markers in patients with SSNHL. However, serological findings alone are insufficient to establish active infection or reactivation.

We identified patients with positive IgM for herpes simplex virus and SARS-CoV-2. In addition, cases compatible with Cytomegalovirus (CMV) reactivation were observed serologically, and notably, one adult patient presented CMV positivity on PCR testing, a finding rarely reported in the literature. These observations suggest that both primary infections and viral reactivation may be associated with the onset and clinical course of SSNHL, although they do not establish a causal relationship.^16^^,^^19^

Despite the absence of confirmed active or reactivated infections in some patients, elevated IgG titers for VZV and EBV were consistently observed, indicating prior immune stimulation and a possible role in susceptibility or recovery dynamics.^20^

PCR testing yielded predominantly negative results, which is consistent with the known limitations of blood-based PCR for neurotropic viruses. HSV, VZV, EBV, and CMV exhibit tropism for cochlear and neural tissues, where viral replication may not be adequately reflected in peripheral blood. Additionally, the interval between symptom onset and sample collection, up to 30 days, may have reduced detectability. Therefore, PCR negativity should not be interpreted as exclusion of viral participation, but rather as a limitation of systemic sampling in inner ear disease.

MRI examinations were performed primarily to exclude retrocochlear pathology. Neuroimaging findings provided additional context for a possible infectious-inflammatory process,^21^ although several methodological limitations must be acknowledged. Imaging was performed using a conventional 1.5-Tesla protocol without dedicated endolymphatic hydrops sequences, reducing sensitivity for detecting subtle inner ear abnormalities. Moreover, in most cases, imaging occurred only after completion of systemic corticosteroid therapy and at delayed intervals following SSNHL onset. This timing may have reduced the ability to identify acute cochlear inflammation, as corticosteroids can suppress contrast enhancement, while enhancement observed later may reflect post-inflammatory changes rather than active disease.

Inner ear signal abnormalities suggestive of inflammatory changes were identified in a subset of patients, more frequently among those with positive viral serology or PCR; however, given the observational nature of the study, these findings are not diagnostic and should be interpreted as indicative of a possible infectious-inflammatory process rather than establishing a causal relationship or defining the underlying etiology of SSNHL.

When considered alongside contemporary literature, including post-COVID SSNHL reports and the prior findings,^16^ our results are consistent with multiple mechanisms for virus-associated auditory dysfunction: (1) Viral reactivation from latency; (2) Endothelial injury and disruption of cochlear microvasculature; (3) Microthrombosis; (4) Cytokine-mediated inflammation; and (5) Direct or immune- mediated damage to the organ of Corti. The observation of coinfections further supports the possibility that combined viral and inflammatory pathways may contribute to cochlear injury and influence recovery.

These data support the concept that SSNHL is a heterogeneous condition in which viral exposure may act as a contributing or modulating factor rather than a direct etiological agent. Serological and imaging findings may therefore be considered complementary tools for clinical characterization and prognostic stratification, although their role in routine clinical decision-making remains to be established.

The present study has limitations that should be acknowledged. Its relatively small sample size, single-center design, and the absence of a control group restrict generalizability and limit causal inference regarding viral association. The absence of a control group also limits interpretation of whether seropositivity exceeds background population levels.

This study was designed as a prospective cohort with consecutive inclusion and longitudinal follow-up of patients with SSNHL, including repeated clinical, audiometric, serological, and imaging assessments during the acute and subacute phases. As such, the focus was on within-cohort temporal and prognostic associations rather than comparison with an external control group.

The interpretation of serological findings remains challenging, and blood-based PCR has inherently low sensitivity for neurotropic viruses.

Comorbidities such as hypertension and diabetes were actively managed during the study period, including monitoring blood pressure and glycemic levels, particularly during corticosteroid therapy. However, standardized longitudinal data allowing formal classification of disease control (e.g., well-controlled vs. uncontrolled) were not consistently available for all patients, which represents a limitation.

Furthermore, the 60–90 day follow-up period was intended to capture early hearing recovery after initial treatment, which occurs predominantly within the first month. Although longer follow-up is relevant for identifying recurrence and other etiologies, this study focused on acute-phase outcomes and infectious associations.

Despite these limitations, the study retains important methodological strengths, including its prospective design and the integration of audiometry, MRI, serology, and PCR. By evaluating both herpesviruses and SARS-CoV-2 during a unique epidemiological period, this study contributes to the growing body of literature exploring potential viral and immunological factors in SSNHL and highlights the need for future multicenter and mechanistic investigations.

## Conclusion

Serological evidence of viral exposure was observed in a substantial proportion of patients with Sudden Sensorineural Hearing Loss (SSNHL). Positivity for herpesviruses and SARS-CoV-2, particularly in cases of coinfection, was associated with poorer clinical outcomes. Initial hearing loss severity and bilateral involvement remained the main prognostic determinants. Intralabyrinthine abnormalities on magnetic resonance imaging were more frequently observed among seropositive patients, suggesting a potential role of virus associated inflammatory mechanisms in SSNHL.

## ORCID ID

Tracy Lima Tavares Soeiro: 0000-0003-0665-2539

Marina Cançado Passarelli Scott: 0000-0002-1854-6509

Breno Lima de Almeida: 0000-0002-3276-617X

Jônatas Bussador do Amaral: 0000-0001-5819-0474

Elizabeth Cristina Perez: 0000-0003-3038-9902

Sujana S. Chandrasekhar: 0000-0002-8523-1769

Norma de Oliveira Penido: 0000-0003-3496-410X

## Authors’ contributions

Tracy Lima Tavares Soeiro: Study design; data acquisition; drafting the manuscript; approval of final version; agree to be held accountable for all information described in the manuscript. Marina Cançado Passarelli Scott and Breno Lima de Almeida: Data acquisition; statistical analysis; approval of final version; critical revision of the final version; agree to be held accountable for all information described in the manuscript. Jônatas Bussador do Amaral and Elizabeth Cristina Perez : Data acquisition; statistical analysis; drafting of the manuscript; critical revision and approval of final version; agree to be held accountable for all information described in the manuscript. Sujana S. Chandrasekhar: Study design; critical revision and approval of final version; agree to be held accountable for all information described in the manuscript. Norma de Oliveira Penido: Study design; data acquisition; drafting the manuscript; critical revision, and approval of final version; agree to be held accountable for all information described in the manuscript.

## Funding

This work was supported by Fundação de Amparo à Pesquisa do Estado de São Paulo (FAPESP; process number 2021/07346-6). Tracy Lima Tavares Soeiro received funding from the Coordination for the Improvement of Higher Education Personnel (CAPES). Norma de Oliveira Penido received funding from the National Council for Scientific and Technological Development (CNPq; process number 402014/2025-1). For the remaining authors, none were declared.

## Data availability statement

The authors declare that all data are available in repository.
